# Myocardial stretch-induced compliance is abrogated under ischemic conditions and restored by cGMP/PKG-related pathways

**DOI:** 10.3389/fphys.2023.1271698

**Published:** 2023-10-02

**Authors:** André M. Leite-Moreira, João Almeida-Coelho, João S. Neves, Ricardo Castro-Ferreira, Ricardo Ladeiras-Lopes, Adelino F. Leite-Moreira, André P. Lourenço

**Affiliations:** ^1^ Cardiovascular R&D Centre—UnIC@RISE, Department of Surgery and Physiology, Faculty of Medicine of the University of Porto, Porto, Portugal; ^2^ Department of Anesthesiology, Centro Hospitalar Universitário São João, Porto, Portugal; ^3^ Department of Endocrinology, Metabolism and Diabetes, Centro Hospitalar Universitário São João, Porto, Portugal; ^4^ Department of Vascular Surgery, Centro Hospitalar de Vila Nova de Gaia/Espinho, Vila Nova de Gaia, Portugal; ^5^ Department of Cardiothoracic Surgery, Centro Hospitalar Universitário São João, Porto, Portugal

**Keywords:** myocardial ischemia, myocardial infarction, cardiac stretch, volume loading, myocardial compliance, diastolic function, phosphodiesterase-5 inhibitors, cGMP-dependent protein kinase

## Abstract

**Introduction:** Management of acute myocardial infarction (MI) mandates careful optimization of volemia, which can be challenging due to the inherent risk of congestion. Increased myocardial compliance in response to stretching, known as stretch-induced compliance (SIC), has been recently characterized and partly ascribed to cGMP/cGMP-dependent protein kinase (PKG)-related pathways. We hypothesized that SIC would be impaired in MI but restored by activation of PKG, thereby enabling a better response to volume loading in MI.

**Methods:** We conducted experiments in *ex vivo* rabbit right ventricular papillary muscles under ischemic and non-ischemic conditions as well as pressure–volume hemodynamic evaluations in experimental *in vivo* MI induced by left anterior descending artery ligation in rats.

**Results:** Acutely stretching muscles *ex vivo* yielded increased compliance over the next 15 min, but not under ischemic conditions. PKG agonists, but not PKC agonists, were able to partially restore SIC in ischemic muscles. A similar effect was observed with phosphodiesterase-5 inhibitor (PDE5_i_) sildenafil, which was amplified by joint B-type natriuretic peptide or nitric oxide donor administration. *In vivo* translation revealed that volume loading after MI only increased cardiac output in rats infused with PDE5_i_. Contrarily to vehicle, sildenafil-treated rats showed a clear increase in myocardial compliance upon volume loading.

**Discussion:** Our results suggest that ischemia impairs the adaptive myocardial response to acute stretching and that this may be partly prevented by pharmacological manipulation of the cGMP/PKG pathway, namely, with PDE5_i_. Further studies are warranted to further elucidate the potential of this intervention in the clinical setting of acute myocardial ischemia.

## 1 Introduction

In acute myocardial infarction (MI) and coronary syndromes, ischemia impairs both systolic and diastolic function (Ratshin et al., 1972). Early revascularization is critical and should not be delayed. Nevertheless, many patients develop cardiogenic shock, and their hemodynamics must be supported (Gowda et al., 2008). Current medical focus has shifted toward mechanical circulatory support, inotropic or vasopressor therapy, and advanced hemodynamic monitoring (Kirigaya et al., 2023), but the first support measure remains fluid resuscitation. Optimization of filling pressures by volume loading increases the cardiac output and allows downtitration of inotropes, thereby preventing side effects (Gowda et al., 2008). Still, the therapeutic margin may be narrow since the acutely ischemic heart already faces elevated filling pressures and there is an impending risk of lung congestion. A deeper understanding of the mechanisms of adjustment to volume loading in physiology and disease becomes crucial.

In its healthy state, the heart swiftly adapts to acute bouts of stretching or overload throughout life. While the mechanisms of systolic function adaptation to stretch have been widely investigated (Neves et al., 2016), diastolic adaptation under physiological conditions has only recently been described in multiple mammalian species and experimental conditions by our group (Leite-Moreira et al., 2018). This adaptive stretch-induced compliance (SIC) response was partly ascribed to titin phosphorylation by cyclic guanosine monophosphate (cGMP)/cGMP-dependent protein kinase (PKG)-related pathways and shown to be decreased in the hypertrophic heart of rats with transverse aortic constriction. We had also previously observed impaired slow force response after stretching rabbit papillary muscles *ex vivo* under ischemic conditions, which could be partly mitigated by the cGMP-specific type 5 phosphodiesterase inhibitors (PDE5_i_) (Castro-Ferreira et al., 2014). Together, these findings led us to hypothesize that SIC might be impaired under ischemic conditions and that pinpointing and targeting the underlying mechanisms might enable broadening the therapeutic margin for volume loading in acute MI.

In this study, we aimed to dissect the effects of stretching myocardial compliance under ischemic conditions *ex vivo*, to elucidate the underlying signaling pathways, and then modulate them *in vivo* during volume loading in experimental acute MI.

## 2 Materials and methods

### 2.1 Ethics and animal care

Animal experiments were approved by the competent authorities (016531) and complied with the Guide for the Care and Use of Laboratory Animals (NIH Publication no. 85–23, revised 2011) and the guidelines from Directive 2010/63/EU of the European Parliament on the protection of animals used for scientific purposes. Male 11-week-old Wistar Han rats (*n* = 14; Charles River, Spain) and 10- to 12-week-old New Zealand white rabbits (*n* = 42) were housed in groups of 3 and 1 per cage, respectively, in a controlled environment at 22°C under a 12:12-h light–dark cycle and with free access to food and water.

### 2.2 Papillary muscle preparations

Male New Zealand white rabbits were anesthetized with intravenous sodium pentobarbital (25 mg kg^−1^) injected through a superficial ear vein. After confirming the loss of toe pinch and ear prick reflexes, a left thoracotomy was performed, and the beating hearts were quickly excised and immersed in modified Krebs–Ringer buffer (KRB; in mM: 98 NaCl, 4.7 KCl, 2.4 MgSO_4_, 1.2 KH_2_PO_4_, 4.5 glucose, 1.8 CaCl_2_, 17 NaHCO_3_, 15 sodium pyruvate, 5 CH_3_COONa, and 30 2,3-butanedione monoxime—BDM) at 30°C with 5% newborn calf serum. Right ventricle (RV) papillary muscles were dissected and vertically mounted in a 10-mL plexiglass organ bath. One or two papillary muscles were used from each animal. The lower muscular end was fixed in a phosphor-bronze clip, the upper tendinous end was attached to an electromagnetic length–tension transducer (University of Antwerp, Belgium), and preload was initially set to 3 mN. After replacement with KRB without calf serum or BDM, the preparations were stimulated at 0.2 Hz with square 5-ms pulses, with the voltage set 10% above the stimulation threshold. The length for maximum active force development was determined (L_max_), and the muscles were allowed to stabilize at 92% of L_max_. The pH was set at 7.4 with 5% CO_2_. The experimental protocol consisted of serial evaluation of passive tension in isometric twitches for 15 min after sudden stretch from 92% to 100% of L_max_. Papillary muscles were randomized to undergo either evaluation under oxidative conditions with 95% O_2_ (*n* = 9) or under conditions mimicking myocardial ischemia (glucose was removed from the bathing solution and O_2_ was interrupted, *n* = 7). Under oxidative (non-ischemic) conditions, further randomization was carried out between no pharmacological manipulation or previous addition of a PKA inhibitor (KT-5720; Sigma-Aldrich; 10^−5^ M; *n* = 7), a PKG inhibitor (Rp-8-Br-PET-cGMPS; Sigma-Aldrich; 10^−6^ M; *n* = 7), or a PKC inhibitor (chelerythrine; Sigma-Aldrich; 10^−5^ M; *n* = 7). Likewise, under ischemic conditions, muscles were randomized to no pharmacological modulation or previous incubation with a PKG agonist (8-bromo-cGMP; Sigma-Aldrich; 10^−5^ M; *n* = 7) or a PKC agonist (phorbol 12-myristate 13-acetate; Sigma-Aldrich; 5 × 10^−6^ M; *n* = 7). Based on the modification of response under ischemic conditions with PKG agonists, new experiments were conducted by randomly assigning muscles to incubation with either B-type natriuretic peptide-32 (BNP; Sigma-Aldrich; 10^−6^ M; *n* = 7), a nitric oxide (NO) donor (S-nitroso-N-acetylpenicillamine (SNAP; Sigma-Aldrich; 10^−5^ M; *n* = 7), or a phosphodiesterase-5 inhibitor (PDE_i_; sildenafil; Pfizer; 10^−6^ M; *n* = 7), and later also with the combinations sildenafil + BNP (*n* = 7) and sildenafil + SNAP (*n* = 6). The doses of inhibitor and agonist drugs were based on supramaximal effects, as reported in previous studies, and following the manufacturers’ recommendations. Drug efficacy was confirmed in previous experiments carried out at the department. Recording and analysis were performed with dedicated software (University of Antwerp, Belgium).

### 2.3 Volume loading after myocardial infarction *in vivo*

Eleven-week-old male Wistar Han rats weighing 416 ± 110 g (*n* = 14) were anesthetized by inhalation of 8% sevoflurane and 100 μg/kg subcutaneous injection of fentanyl. Upon orotracheal intubation with a 14G intravenous catheter, anesthesia was maintained with 2.5%–3% sevoflurane, and animals were mechanically ventilated (PhysioSuite^®^, Kent Scientific) with 100% O_2_ in a volume-controlled mode with end-expiratory pressure held at 4 cmH_2_O. The respiratory rate and tidal volume were adjusted to achieve normocapnia. Body temperature was kept at 37–38°C with a heating pad. Peripheral venous access (24G) was placed in the dorsal foot vein, and warm Ringer lactate solution was infused at a rate of 20 mL/kg/h. Surface electrocardiogram (ECG), peripheral oximetry, and body temperature were monitored throughout the procedure. In the position of a slight right lateral decubitus and upon wide left thoracotomy, a pressure–volume catheter was placed along the left ventricular long axis through an apical puncture (SPR-869, Millar), a pressure catheter was placed in the pulmonary artery through a puncture in the RV outflow tract (PVR-1045, Millar), and a transit-time flow probe (MA-2.5PSB, Transonic) was placed in the ascending aorta. When the preparation was deemed stable, rats were subjected to acute MI by left anterior descending artery ligation with a 5-0 polypropylene suture 2–3 mm distal to the anterior–inferior edge of the left auricle following a line connecting the intersection between the RV outflow tract and the right border of the left auricle and the left ventricular apex (Hou et al., 2011). MI was confirmed by ST segment changes and arrhythmia development on the ECG, hemodynamic changes, and by visual confirmation of dyskinesia and pallor of the left ventricular anterior wall (illustrative video and ECG changes are presented in Supplementary Material). When rhythm and hemodynamics were deemed stable, rats were randomized to either sildenafil 42.5 μg/kg/min or an equal volume of infused vehicle (groups Sil and Veh, *n* = 7 each, respectively), and after at least 20 min of infusion, upon stabilization, Veh and Sil were then subjected to acute volume loading. Volume loading consisted of an infusion of 10% estimated extracellular fluid volume for 15 min. Extracellular water was estimated as 24% of body weight (Cornish et al., 1992). The solution infused was a 50% mixture of 20% mannitol and Ringer lactate, with one-fourth of the volume infused in 2 min and maintenance and the remaining three-fourths for the next 13 min.

There was no mortality, and ventricular fibrillation episodes were resolved by transient cardiac massage and defibrillation by a sudden flick of the myocardium with the back of a metal forceps. All signals were continuously recorded at 2000 Hz (MPVS ultra, LabChart Pro, ADInstruments). Inferior vena cava occlusions with ventilation suspended at end-expiration were carried out at baseline, after MI, upon drug/vehicle infusion and, finally, after volume loading. Pressure–volume signals were calibrated by aortic flow (slope factor α calibration) and repeated injection of 40 µL 10% hypertonic saline (parallel conductance). End-systolic and end-diastolic pressure–volume relationships were fitted as end-systolic elastance (E_es_)*(end-systolic volume—zero volume (v0)) and α*e^end-diastolic volume*β^ upon confirmation that the non-linear component and pressure decay asymptote were neglectable, respectively.

### 2.4 Statistical analysis

Pressure–volume signal analysis was performed by Python scripts developed in-house, and statistical analysis was carried out with statsmodels and pingouin mixed_anova Python packages for two-way repeated measures analysis of variance, except for the end-systolic and end-diastolic pressure–volume relationships, which were analyzed with tydiverse and rstatix R packages for two-way repeated measures analysis of covariance. Plots were generated with the matplotlib Python package. Experiments conducted on papillary muscles were analyzed with two-way repeated measures (passive tension evolution in ischemic and non-ischemic myocardium) or one-way analysis of variance (comparisons of percent passive tension decay between experimental groups). Assumptions were checked by visual inspection of model residuals and Shapiro–Wilk’s test, Levene’s test for homogeneity of variances, Mauchly’s test for sphericity assumption, and Box-M test for homogeneity of covariates. There were no major deviations from assumptions: when sphericity assumption was not met, the Greenhouse–Geisser correction was applied. Multiple groups’ pairwise comparisons were performed with Sidak’s adjustment. Data are expressed as mean ± standard error of mean. The significance value ascertained by the two-tailed test was set at 0.05.

## 3 Results

### 3.1 Response to stretch in non-ischemic and ischemic myocardium

**FIGURE 1:**
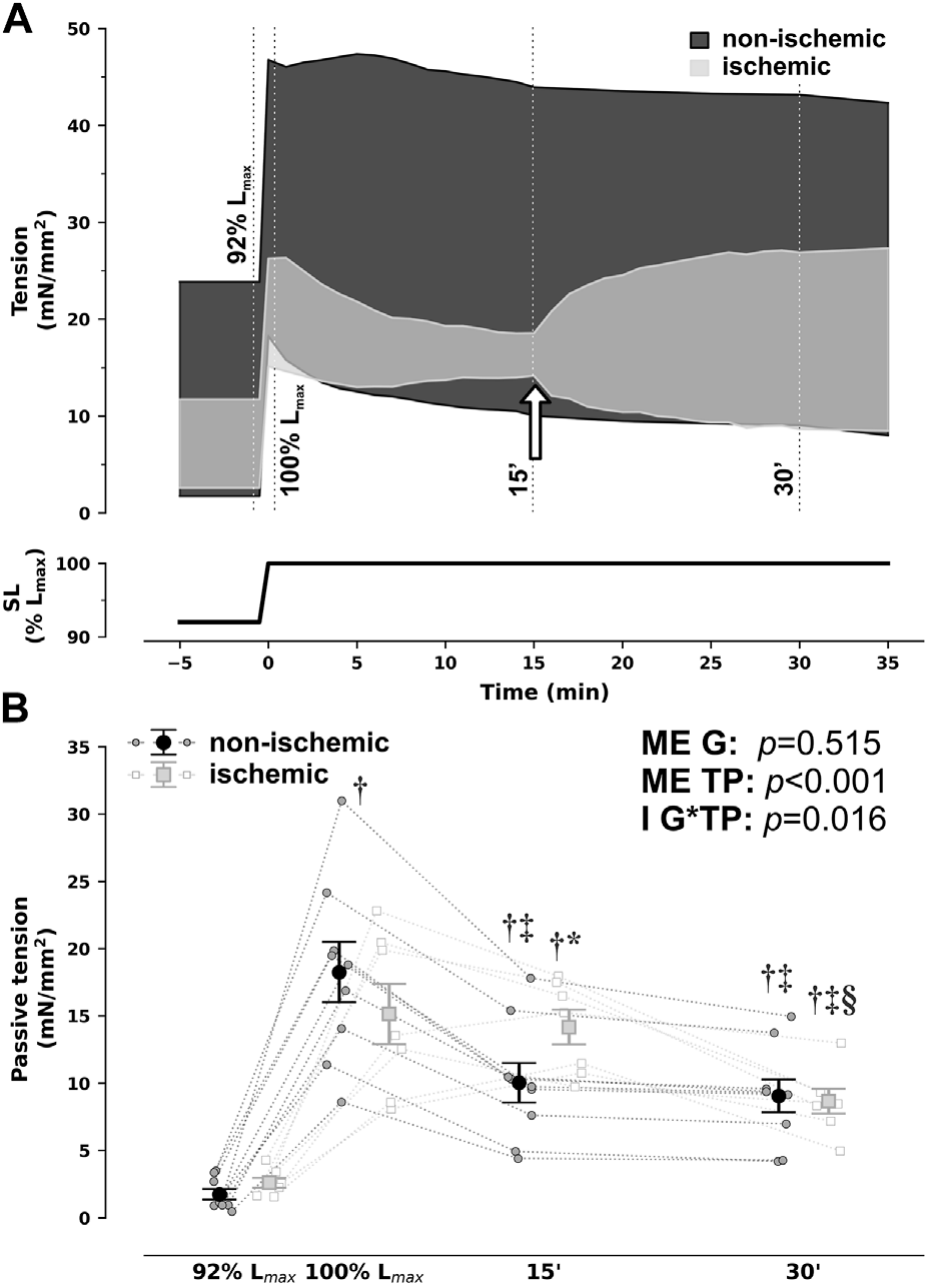
Group-averaged tracings of developed tension in isometric contractions of right ventricular rabbit papillary muscles under non-ischemic and ischemic conditions when sudden and sustained stretch from 92% to 100% of optimal maximum muscle and sarcomere length (SL, L_max_) was applied **(A)** and passive tension comparisons at selected timepoints **(B)**. For illustration purposes, the timepoints of evaluation from **(B)** are depicted by dotted lines in **(A)**. In addition, in **(A)**, the white arrow signals denote changing perfusion conditions from ischemic back to non-ischemic in the ischemic group. Ischemic and non-ischemic muscles showed a comparable increase in passive tension upon stretch, but while non-ischemic muscles developed the expected Frank–Starling and slow-force response and a steady decrease in passive tensions throughout the next 15 min (stretch-induced compliance), ischemic muscles did not. Nonetheless, passive tension did decrease after restoration of non-ischemic conditions from 15 to 30 min *p < 0.05 *vs* non-ischemic (same timepoint), ^†^ *p* < 0.05 vs. 92% L_max_, ^‡^ *p* < 0.05 vs. 100% L_max_, ^§^ *p* < 0.05 *vs* 15′ by two-way repeated measures analysis of variance; *n* = 9 and 7 for non-ischemic and ischemic, respectively. G, group; I, interaction; ME, main effect, TP, timepoint.

As previously reported, rabbit RV papillary muscles steadily decreased passive tension from 92% to 100% of Lmax under non-ischemic conditions, in the 15 min that followed sudden stretch, denoting SIC (Figure 1). This response was entirely abrogated in ischemia but fully restored upon return to aerobic conditions, as can be clearly visualized in Figure 1A.

**FIGURE 2:**
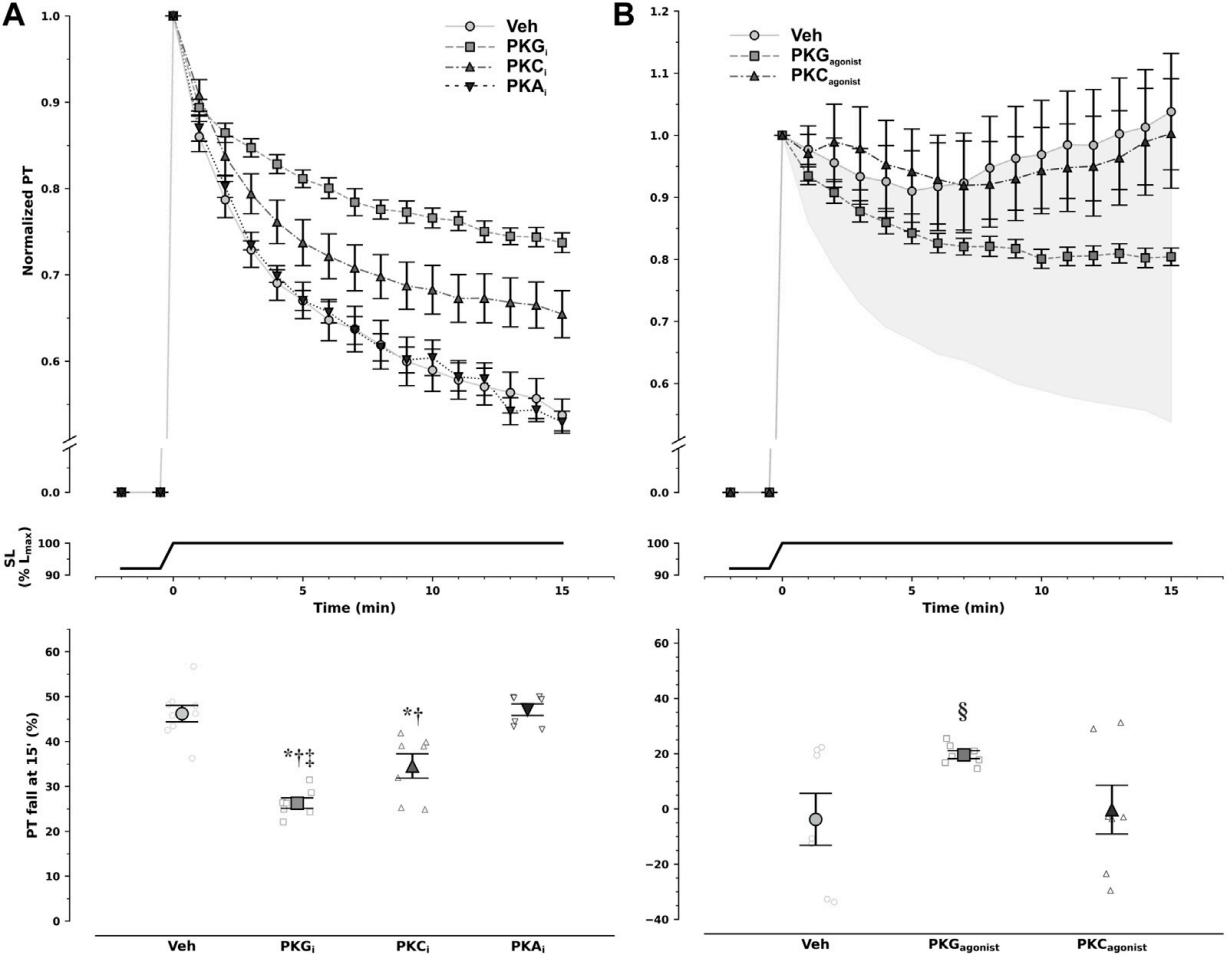
Modulation of diastolic response to stretch by protein kinase (PK) pathways. Non-ischemic muscles were incubated with either vehicle (Veh) or PK inhibitors **(A),** whereas ischemic muscles were incubated with either vehicle (Veh) or PK agonists **(B)**. Decay of normalized passive tension (PT) during the 15 min that followed stretch from 92% to 100% of optimal maximum muscle and sarcomere length (Lmax, SL) is presented at the top, while overall magnitude of PT decrease is shown at the bottom. Inhibition of PKA (PKAi) had no effect on passive tension decrease, whereas PKC inhibition (PKCi) slightly reduced it, and PKG inhibition (PKGi) attenuated it by approximately 50% in non-ischemic muscles. Under ischemic conditions only PKG, and not PKC, activation was able to partly restore passive tension decrease with stretch (^§^ *p* = 0.029 vs. Veh and PKC by least-squares contrasts). In panel B, the shaded area represents the difference in magnitude of response between ischemic and non-ischemic myocardium. Remarkably, PKG agonism restored nearly 50% of pressure decay in the ischemic myocardium. PKA agonists were not tested. Comparisons were performed with one-way analysis of variance: ^*^ *p* < 0.001 *vs* Veh, ^†^ *p* < 0.001 vs. PKA_i,_ and ^‡^ *p* = 0.026 vs. PKC_i_; *n* = 9, 7, 7, and 7 for Veh, PKGi, PKCi, and PKAi in non-ischemic muscles, respectively, and 7, 7, and 7 for Veh, PKG agonism, and PKC agonism in ischemic muscles, respectively.

To dissect the underlying intracellular pathways, we probed the contributions of protein kinases PKA, PKC, and PKG by previous incubation with direct inhibitors in non-ischemic muscles. PKG inhibition substantially reduced PT to approximately half. PKC inhibition also slightly blunted PT decrease, while inhibition of PKA had no effect (Figure 2A).

### 3.2 Restoring stretch-induced compliance in ischemic myocardium *ex vivo*

PKC activation had no effect on PT decay after stretching under ischemic conditions, whereas PKG inhibition partly restored the response (Figure 2B). Noticeably, inhibition and stimulation of PKG in non-ischemia and ischemia, respectively, had symmetric effects, implying not only PKG pathway involvement in SIC but also the potential to restore the healthy response in ischemia. Of note, given the previous results, stimulation of PKA in ischemia was not further pursued.

**FIGURE 3:**
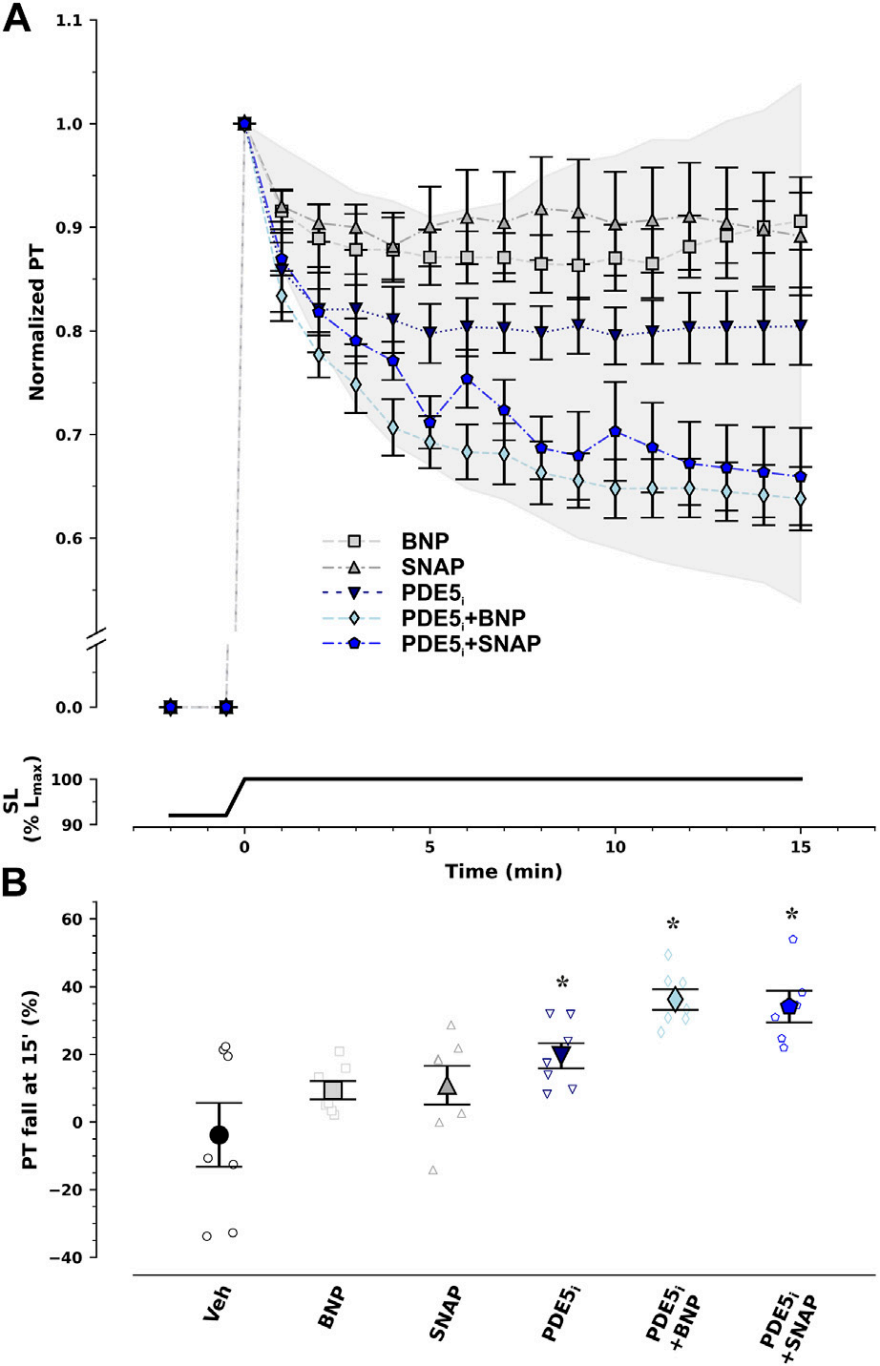
Recovery of response to stretch ex vivo under ischemic conditions by pharmacological modulation of the protein kinase G/cyclic guanosine monophosphate pathway. Normalized passive tension (PT) decay after stretch from 92% to 100% of optimal maximum muscle and sarcomere length (SL, L_max_) is presented in **(A)**, while final percentage of PT decrease at 15 min is summarized in **(B)**. Type-B natriuretic peptide (BNP) and nitric oxide (NO) donor S-nitroso-N-acetylpenicillamine (SNAP) had no effect. On the other hand, type 5 phosphodiesterase inhibitor (PDE5_i_) sildenafil restored PT decay by 50% and combination therapy with PDE5_i_ + BNP or PDE5_i_ + SNAP almost completely reestablished PT decay with stretch. The extent of the effect can be appraised from the shaded area which represents the difference in response between ischemic and non-ischemic myocardium. *p < 0.05 *vs* Veh by one-way analysis of variance; *n* = 7 in each group, except for PDE5_i_ + SNAP (*n* = 6).

To further exploit the PKG pathway, we undertook incubation with several modulators of this pathway, namely, natriuretic peptides, NO donors, and the PDE5_i_ sildenafil (Figure 3). In isolation, neither BNP nor SNAP restored response to stretch under ischemic conditions. Contrastingly, PDE5_i_ recovered it by nearly half. Nevertheless, only the combination of PDE5_i_ with either natriuretic peptide or an NO donor enabled almost full restitution of the effect.

### 3.3 Translation to *in vivo*: restoring stretch-induced compliance with PDE5i in acute myocardial infarction

The summary of hemodynamic data is presented in Table 1. Despite randomization, rats that underwent sildenafil infusion showed a trend toward higher heart rates and faster relaxation, as appraised by the time-constant of isovolumetric relaxation, ***τ***, and higher peak pressure derivative values at baseline. Nevertheless, there were no differences between groups regarding cardiac output, end-diastolic volume, and load-independent indexes of left ventricular contractility or compliance.

**TABLE 1:** Summary of pressure–volume data.

	Baseline		MI		Drug/vehicle		Volume loading		ME	ME	Interaction
	Veh	Sil	Veh	Sil	Veh	Sil	Veh	Sil	G	TP	G * TP
HR, bpm	349 ± 12	376 ± 14	354 ± 14	383 ± 16	348 ± 12	408 ± 12^*†‡^	325 ± 10^‡^	381 ± 9^*§^	0.025	0.011	0.040
CO, mL/min	39 ± 1	42 ± 2	39 ± 2	41 ± 2	41 ± 3	42 ± 1	44 ± 4	57 ± 2^*†‡§^	0.200	<0.001	0.004
mPAP, mmHg	15 ± 1	14 ± 2	14 ± 1	13 ± 1	15 ± 1	15 ± 1	17 ± 2^†‡^	14 ± 2^*^	0.564	0.045	0.269
EF, %	66 ± 3	58 ± 1	56 ± 2	47 ± 2^*†^	49 ± 1	54 ± 3	45 ± 3^†^	55 ± 2^*^	0.817	<0.001	0.003
P_max_, mmHg	115 ± 2	124 ± 3	104 ± 2^†^	117 ± 4^†^	101 ± 1^†^	109 ± 3^†^	112 ± 6	114 ± 3	0.058	0.002	0.291
EDP, mmHg	6 ± 1	5 ± 0	10 ± 1^†^	10 ± 1^†^	9 ± 1	8 ± 1^†‡^	16 ± 2^†‡§^	11 ± 1^†^	0.437	<0.001	0.021
EDV, μL	174 ± 13	190 ± 7	201 ± 13^†^	209 ± 5^†^	232 ± 16^†^	200 ± 12	300 ± 22^†‡§^	288 ± 17^†‡§^	0.758	<0.001	0.103
dP/dt_max_, mmHg/s	6,470 ± 183	7,380 ± 191^*^	5,950 ± 220^†^	6,910 ± 221^*†^	5,800 ± 156^†^	6,940 ± 176^*†^	6,270 ± 304	7,380 ± 179^*^	<0.001	0.004	0.842
*τ*_logistic_, ms	7.2 ± 0.5	6.0 ± 0.2	8.0 ± 0.6	6.2 ± 0.3^*^	7.8 ± 0.6	5.3 ± 0.3^*^	8.0 ± 0.7	5.5 ± 0.2^*^	0.004	0.275	0.161
*β*,/mL	10.3 ± 2.91	12.6 ± 0.77	11.7 ± 2.68	10.7 ± 1.89	13.1 ± 3.34	10.7 ± 1.52	13.9 ± 2.19	5.65 ± 1.31^*^	0.001	0.192	0.77
e_es_, mmHg/μL	3.1 ± 0.6	4.6 ± 0.7	2.9 ± 0.7	4.3 ± 0.5	2.0 ± 0.3	4.4 ± 0.6^*^	1.8 ± 0.3	2.8 ± 0.4	<0.001	0.030	0.729
PRSW, mmHg	105 ± 9	95 ± 7	48 ± 3^†^	58 ± 6^†^	57 ± 4^†^	61 ± 4^†^	50 ± 4^†^	64 ± 4^*†^	0.376	<0.001	0.127

Summary of *in vivo* hemodynamic pressure–volume data recorded sequentially at baseline, after myocardial infarction (MI) induction, after vehicle (Veh) or drug (sildenafil) administration (Sil), and, finally, after subsequent volume loading. HR, heart rate; CO, cardiac output; mPAP, mean pulmonary artery pressure; EF, ejection fraction; P_max_, maximum pressure; EDP, end-diastolic pressure; EDV, end-diastolic volume; dP/dt_max_, peak (maximum) pressure derivative; *τ*_logistic_, time constant of isovolumetric relaxation by the logistic method; *β*, chamber stiffness constant derived from the end-diastolic pressure–volume relationship; e_es_, end-systolic elastance; PRSW, preload recruitable stroke work; *p < 0.05 *vs* other group at the same timepoint, ^†^ *p* < 0.05 *vs* baseline, ^‡^ *p* < 0.05 *vs* MI, ^§^ *p* < 0.05 *vs* drug/vehicle with two-way repeated-measures analysis of variance; *n* = 7 for each group. For β and e_es_ analysis, we included α and v0 as covariates, respectively (analysis of covariance). The main effect (ME) for group (G), timepoint (TP), and their interaction are presented in the three rightmost columns.

**FIGURE 4:**
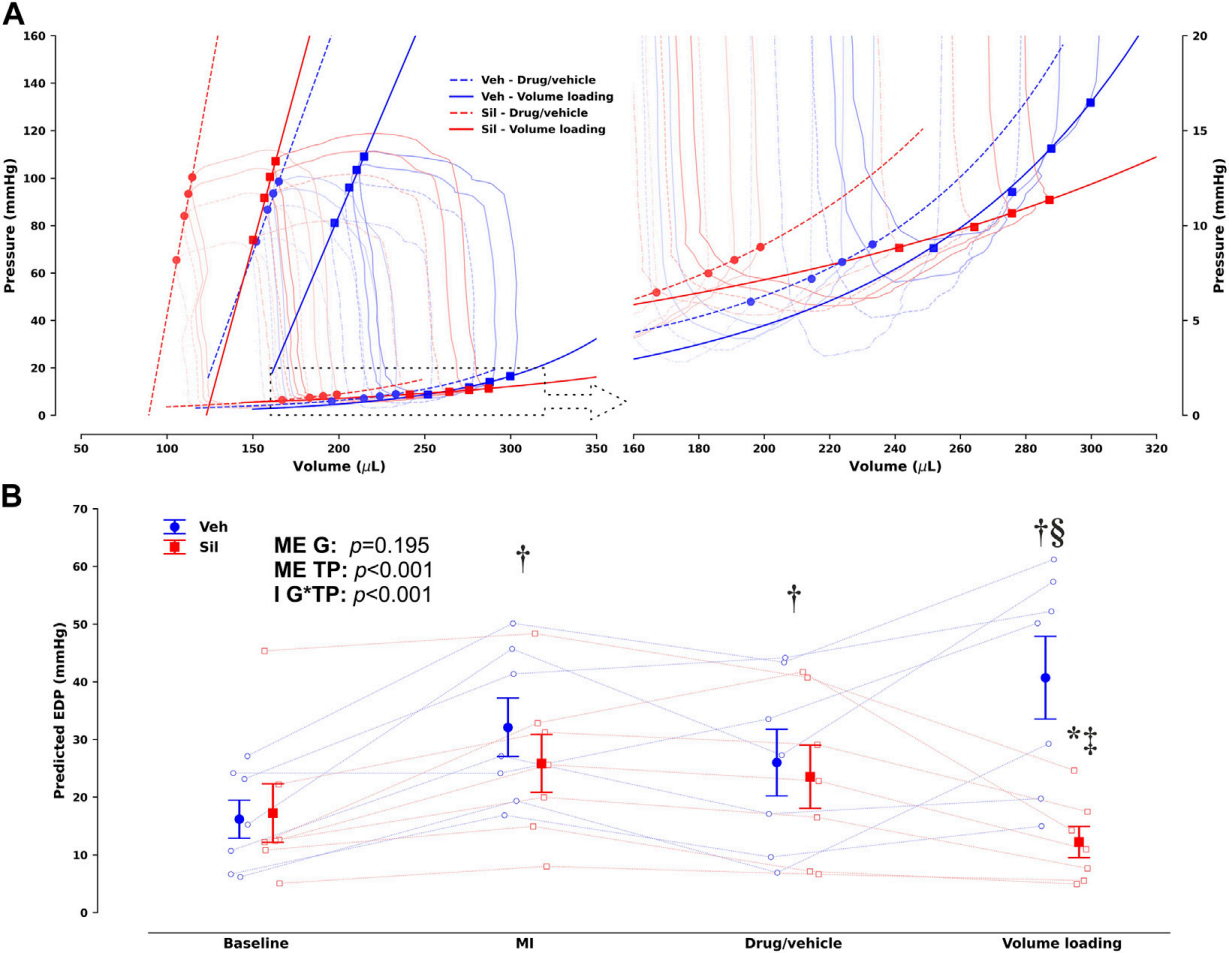
Group- and timepoint-averaged pressure–volume (PV) loops and corresponding end-systolic and end-diastolic PV relationships (EDPVR) before and after volume loading in vehicle-treated (Veh) and sildenafil-treated (Sil) rats upon myocardial infarction induction [**(A)**, with the EDPVR highlighted on the right] and corresponding predicted end-diastolic pressures for a common volume of 350 µL based on the EDPVR fits **(B)**. Unlike **(B),** not all timepoints are presented in **(A)** to avoid image clutter; the timepoint before volume loading is marked by circles and dashed lines, whereas squares and solid lines mark the timepoint after volume loading (drug/vehicle and volume loading, respectively, to retain compatibility with Table 1). A clearly different response to volume loading is evident in Sil, which increases compliance. This difference is easily appraised and quantified in **(B)**, where the main effects (ME) of the group (G) and timepoint (TP) are presented along with the interaction effect (I). *p < 0.05 *vs* other group at the same timepoint, ^†^ *p* < 0.05 *vs* baseline, ^‡^ *p* < 0.05 *vs* MI, ^§^ *p* < 0.05 *vs* drug/vehicle by two-way repeated-measures analysis of variance; *n* = 7 for each group.

Upon MI, both Veh and Sil showed decreased left ventricular maximum pressure (P_max_) and elevated end-diastolic volumes and pressures, as well as a drop in ejection fraction and preload recruitable stroke work (PRSW). No remarkable changes in hemodynamics were observed after vehicle or sildenafil infusion, at least for the duration of the stabilization period. After myocardial stretch by volume loading, as expected, both Veh and Sil exhibited increased end-diastolic volumes. There were remarkable differences between groups, however, in terms of left ventricular function. Compared with Veh, Sil showed not only increased cardiac output and improved systolic function, as appraised by both ejection fraction and load-independent PRSW, but also enhanced myocardial compliance, denoted by a minor increase in end-diastolic pressures and by a decrease in chamber stiffness constant, *β*. For simplicity, representative pressure–volume loops and end-systolic and end-diastolic regressions before and after volume loading are shown in Figure 4A. In Figure 4B, we depict predicted end-diastolic pressures derived according to the fitting parameters per animal and timepoint for a common volume of 350 μL at all timepoints (Panel B). Higher compliance after volume loading in Sil is clearly visualized both by the gentler slope of the end-diastolic pressure–volume relationships (panel A) and by the consistent decrease in predicted end-diastolic pressures (panel B).

## 4 Discussion

In this study, we demonstrate that SIC, the increase in myocardial compliance upon stretching, is abrogated under ischemic conditions. PKG-related pathways not only contribute to this adaptive response in the non-ischemic myocardium but are also able to partly restore it in ischemia *ex vivo*. These findings were translated to *in vivo* experimental MI. Type 5 phosphodiesterase (PDE5) inhibition with sildenafil after MI enhanced myocardial performance, enabling lower filling pressures during volume loading.

### 4.1 The diastolic response to stretching is impaired by myocardial ischemia

Systolic function is enhanced by myocardial stretch through intricate mechanisms that have been extensively investigated in physiology, from the Frank–Starling mechanism to slow-force response (Neves et al., 2016). Given the strong interlinks between systolic and diastolic physiology, it comes as no surprise that diastolic adaptations are also at play. Indeed, we have described SIC (Leite-Moreira et al., 2018), a physiological mechanism that enables the heart to cope with overload by increasing compliance in several experimental preparations, as well as in human volunteers. Like any other physiological response, disturbances are expected in disease conditions. In fact, our preliminary reports suggested impaired SIC in the hypertrophic heart of rats with transverse aortic constriction.

Myocardial ischemia has a strong effect on both systolic and diastolic function. Deprivation of energy stores will not only lead to disturbances in calcium handling and impaired crossbridge cycles but also to abnormal cell-signaling mechanisms (Zhang et al., 2022). Furthermore, an increased ADP/ATP ratio can independently increase myocardial stiffness by increasing the proportion of crossbridges in the bound state (Sequeira et al., 2015). Classic experiments showed increased left ventricular end-diastolic pressure after MI in animals and humans (Hood et al., 1970; Ratshin et al., 1972).

This suggests that the physiological response to loading will most likely be compromised as well. Indeed, early clinical experiments with MI patients in cardiogenic shock revealed that the capacity to respond favorably to fluid challenges strongly predicts better prognosis (Russell et al., 1970; Loeb et al., 1973) while, more recently, abnormal exercise tolerance shortly after acute MI has also been associated with poorer outcomes (Andersen et al., 2012). Our results in *ex vivo* simulated hypoxic conditions clearly demonstrate that SIC is blunted in ischemia.

### 4.2 Stimulation of cGMP/PKG activity restores stretch-induced compliance under ischemic conditions

In our past mechanistic studies of SIC, we have partly ascribed the effects on compliance to titin phosphorylation mediated by upstream cell-signaling pathways, pinpointing PKG-related pathways as the most likely responsible (Leite-Moreira et al., 2018). However, other stretch-activated protein kinases such as PKA and PKC can also target titin in domains that influence its elastic properties, including PKA and PKC (Linke and Hamdani, 2014). By direct pharmacological inhibition, a potential role for PKC, but not PKA, was also unveiled. Still, the most relevant effect could be attributed to PKG. Indeed, in contrast to PKG activation, pharmacological activation of PKC under ischemic conditions had no appreciable effect on myocardial compliance after stretch, something that we had also reported for the slow-force response (Neves et al., 2013). Given the effects of PKG agonism on stretch response in ischemia, we pursued further elucidation of the ability of its upstream activators BNP and NO (as direct stimulators of particulate and soluble guanylyl cyclase, respectively) as well as of PDE5_i_ that prevent degradation of cGMP. Only PDE5_i_ was able to recover SIC, suggesting that preventing degradation of cGMP is a key step to restore PKG activation under ischemic conditions. Moreover, despite the differential subcellular localization of nitric oxide and natriuretic soluble peptide and particulate guanylyl cyclase receptors, along with their pathways being modulated by diversely localized phosphodiesterases (Fischmeister et al., 2006), the addition of BNP or NO donors to PDE5_i_ enhanced the response to the point of near restoration of SIC by the synergistic effect.

### 4.3 Phosphodiesterase-5 inhibition improves load tolerance during acute myocardial infarction

As a final step, we attempted to translate our findings *ex vivo* to the *in vivo* scenario. This translation is of course fraught with many limitations. Although we employed a reproducible model of MI (Hou et al., 2011), we acknowledge that there might have been some heterogeneity in the extent of ischemic areas between groups and that non-uniformity and systemic and neurohumoral responses to MI and sildenafil add another layer of complexity to the issue. Moreover, although restoration of SIC in ischemia was optimal under combination therapy *ex vivo*, for practical purposes and to avoid a marked decrease in systemic vascular resistance, only PDE5_i_ was administered *in vivo*.

PDE5_i_ has well-recognized beneficial effects in MI, namely, a reduction in infarct size (Reffelmann and Kloner, 2003), mitigation of reperfusion injury (Ebner et al., 2013; Andersen et al., 2016), and overall cardioprotection (Botha et al., 2010). Indeed, PDE5_i_’s cardioprotective effects include inhibition of Na^+^/H^+^ exchanger-1, modulation of Ca^2+^ handling, and mitochondrial permeability transition, and many of these may even be independent of PKG (Elrod et al., 2007; Perez et al., 2007; Das et al., , 2008; Inserte and Garcia-Dorado, 2015) or related to cGMP signaling in distinct subcellular compartments of non-cardiomyocytes (Cuello and Nikolaev, 2020). On clinical grounds, in the SIDAM trial (Andersen et al., 2013), sildenafil therapy for 9 weeks increased LV end-diastolic volume and cardiac index after MI, with further enhancement during exercise, and despite the negative results in the primary outcome measure, a drop in pulmonary capillary wedge pressure was shown. Many of these multifarious actions of PDE5_i_ might be at play and partly explain our results.

Nevertheless, we did observe a sharp increase in myocardial compliance after volume loading in MI rats upon sildenafil infusion. This response was clearly different from that of the vehicle group. Together with our *ex vivo* findings, this strongly suggests that PDE5_i_ enhances myocardial compliance in ischemia and thus might broaden the therapeutic margin for volume loading in acute MI. By restoring SIC, PDE5_i_ might enable a more favorable hemodynamic response to volume loading in acute MI, while reducing the risk of lung congestion.

### 4.4 Study limitations

Our results are preclinical and purely experimental. The MI model did not entail cardiogenic shock, and there was no need for inotropic or vasopressor support. There was also no reperfusion, and therefore ischemia–reperfusion injury was not studied. The effects of sildenafil in cardiogenic shock and ischemia–reperfusion injury may be substantially different. Infarct size was also not formally measured. The *ex vivo* experimental protocol emulated ischemia by glucose and O_2_ deprivation, which is a poor substitute for MI. Though invaluable for pharmacological studies at the cellular and tissue level, these preparations significantly deviate from physiology, due to the absence of blood perfusion. Interrupting the O_2_/CO_2_ supply entails not only anaerobic metabolism and acidosis due to hypoxia but also opposite changes in acid–base balance due to the lack of CO_2_ supply. Experiments were carried out in right, and not in the bulkier left, ventricular papillary muscles to avoid core hypoxia. Sympathetic denervation intrinsic to the *ex vivo* setup limits upstream activation of signaling pathways that enhance PKA activity and therefore may influence the apparent absence of the effect of inhibiting this protein kinase on SIC.

## 5 Conclusion

We were able to demonstrate that ischemia impairs the adaptive myocardial response to stretching or volume loading and that this may be partly prevented by pharmacological manipulation of the PKG pathway, namely, with PDE5_i_. Translation to the clinical scenario will require well-designed randomized trials where cautious use of PDE5_i_ and volume loading should be tested in terms of outcomes in acute MI.

## Data Availability

The raw data supporting the conclusion of this article will be made available by the authors, without undue reservation.
