# Supplementary material for: Myocardial stretch-induced compliance is abrogated under ischemic conditions and restored by cGMP/PKG-related pathways
Source: Front Physiol. 2023 Oct 2;14:1271698. doi: 10.3389/fphys.2023.1271698 (PMC10577181; doi:10.3389/fphys.2023.1271698)
Supplement: Supplementary file 1 [file DataSheet1.pdf]

Supplementary video. ***Experimental myocardial infarction preparation***

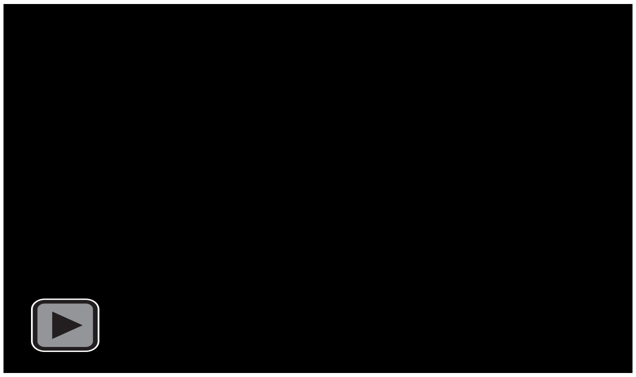

Aortic transit-time flow probe, left ventricular (LV) pressure-volume (PV) and pulmonary artery (PA) catheters are signaled. Both the position of left anterior descending artery ligation by a 6/0 polypropilene suture and the pale dyskinetic area in the anterior LV wall are marked by arrows.

Supplementary figure. ***Electrocardiographic changes upon acute myocardial infarction (MI)***

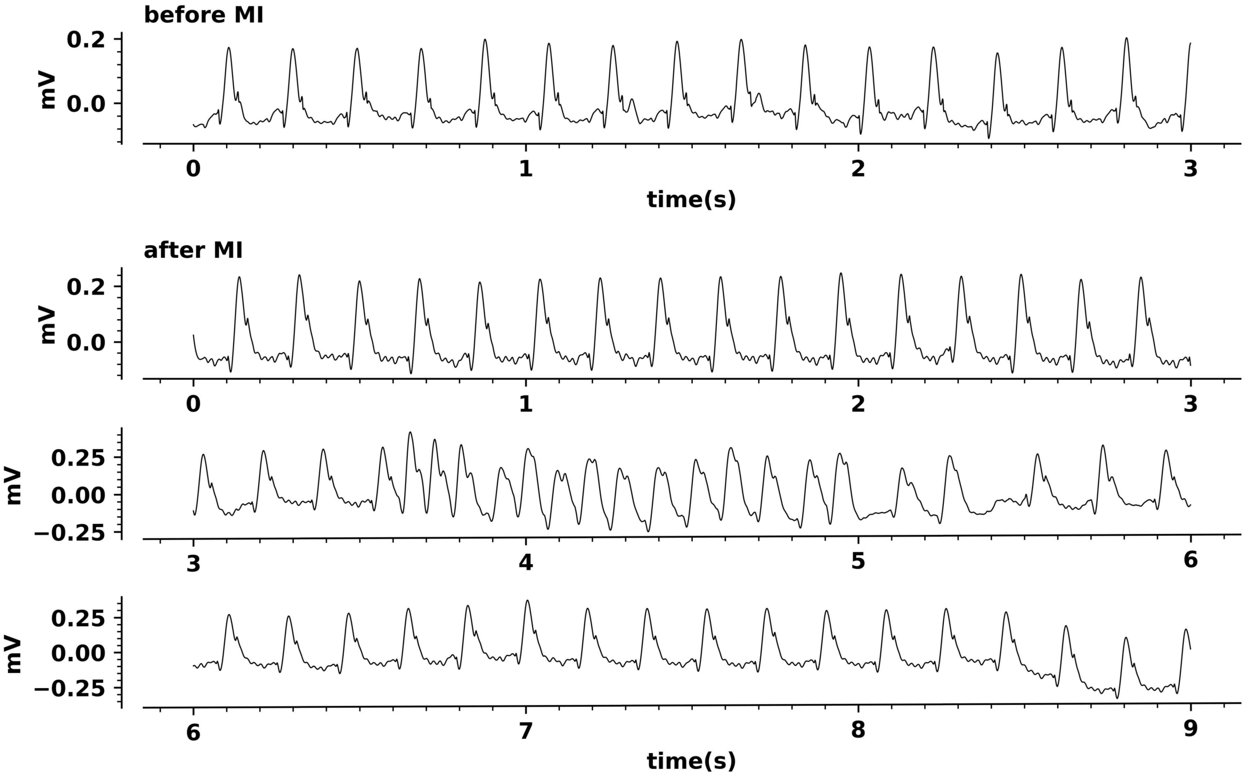

ST-segment elevation is conspicuous after MI. Periods of ventricular arrhythmia were common.
